# Supplementary material for: End Sequence Analysis Toolkit (ESAT) expands the extractable information from single-cell RNA-seq data
Source: Genome Res. 2016 Oct;26(10):1397–410. doi: 10.1101/gr.207902.116 (PMC5052061; doi:10.1101/gr.207902.116)
Supplement: Supplemental Material [file supp_gr.207902.116_Supp_Materials.tar.gz › ESAT-esat_v0.1/doc/html/classbroad_1_1core_1_1motif_1_1_position_weight_column-members.html]

ESAT: Member List


ESAT


- Main Page
- Related Pages
- Packages
- Classes
- Files

- Class List
- Class Index
- Class Hierarchy
- Class Members

All Classes Namespaces Files Functions Variables Enumerator Pages

- broad
- core
- motif
- PositionWeightColumn

broad.core.motif.PositionWeightColumn Member List

This is the complete list of members for broad.core.motif.PositionWeightColumn, including all inherited members.

|  |  |  |
| --- | --- | --- |
| addPseudoCounts() | broad.core.motif.PositionWeightColumn |  |
| computeProb() | broad.core.motif.PositionWeightColumn |  |
| DEFAULT\_ALPHABET\_SIZE | broad.core.motif.PositionWeightColumn | static |
| flatWeight(double infoTh) | broad.core.motif.PositionWeightColumn |  |
| getAlphabetSize() | broad.core.motif.PositionWeightColumn |  |
| getBaseIUB() | broad.core.motif.PositionWeightColumn |  |
| getComplement() | broad.core.motif.PositionWeightColumn |  |
| getInformationContent() | broad.core.motif.PositionWeightColumn |  |
| getLogProb(char ch) | broad.core.motif.PositionWeightColumn |  |
| getLogProb(int baseIdx) | broad.core.motif.PositionWeightColumn |  |
| getLogRatio(char ch) | broad.core.motif.PositionWeightColumn |  |
| getLogRatio(char i, boolean isNumSeq) | broad.core.motif.PositionWeightColumn |  |
| getLogRatioWeightedByNegEntropy(char ch) | broad.core.motif.PositionWeightColumn |  |
| getLogRatioWeightedByNegEntropy(char i, boolean isNumSeq) | broad.core.motif.PositionWeightColumn |  |
| getProbability(int baseIndex) | broad.core.motif.PositionWeightColumn |  |
| getWeight(int letterIndex) | broad.core.motif.PositionWeightColumn |  |
| init(int alphabetSize) | broad.core.motif.PositionWeightColumn |  |
| init() | broad.core.motif.PositionWeightColumn |  |
| kullbackLeiber(PositionWeightColumn pwc) | broad.core.motif.PositionWeightColumn |  |
| PositionWeightColumn() | broad.core.motif.PositionWeightColumn |  |
| PositionWeightColumn(String[] rawData) | broad.core.motif.PositionWeightColumn |  |
| PositionWeightColumn(int[] n) | broad.core.motif.PositionWeightColumn |  |
| PositionWeightColumn(int nA, int nC, int nG, int nT) | broad.core.motif.PositionWeightColumn |  |
| PositionWeightColumn(double[] freq) | broad.core.motif.PositionWeightColumn |  |
| print() | broad.core.motif.PositionWeightColumn |  |
| printProb() | broad.core.motif.PositionWeightColumn |  |
| resetProbWithNullPriorProb() | broad.core.motif.PositionWeightColumn |  |
| resetToUniformProb() | broad.core.motif.PositionWeightColumn |  |
| setFromCounts(int[] n) | broad.core.motif.PositionWeightColumn | protected |
| setNum(int[] n) | broad.core.motif.PositionWeightColumn |  |
| setNum(int nA, int nC, int nG, int nT) | broad.core.motif.PositionWeightColumn |  |
| toConsensus() | broad.core.motif.PositionWeightColumn |  |
| toString() | broad.core.motif.PositionWeightColumn |  |
| write(BufferedWriter bw, NumberFormat formatter) | broad.core.motif.PositionWeightColumn |  |


---

Generated on Fri Aug 15 2014 15:10:54 for ESAT by  

 1.8.7
